# Possible Function of the ribT Gene of Bacillus subtilis: Theoretical Prediction, Cloning, and Expression

**Published:** 2014

**Authors:** A. P. Yakimov, T. A. Seregina, A. A. Kholodnyak, R. A. Kreneva, A. S. Mironov, D. A. Perumov, A. L. Timkovskii

**Affiliations:** B.P. Konstantinov Petersburg Nuclear Physics Institute, National Research Center “Kurchatov Institute”, Orlova Roshcha, Gatchina, Leningrad Region, Russia, 188300; St. Petersburg State Polytechnical University, Polytechnicheskaya Str., 29, St. Petersburg, Russia, 195251; State Research Institute of Genetics and Selection of Industrial Microorganisms, 1st Dorozhnyi Proezd, 1, Moscow, Russia, 117545

**Keywords:** proteomics, bioinformatics, homology search, theoretical protein structure, gene cloning, inducible expression

## Abstract

The complete decipherment of the functions and interactions of the elements of the riboflavin biosynthesis operon (*rib *operon) of *Bacillus subtilis *are necessary for the development of superproducers of this important vitamin. The function of its terminal *rib*T gene has not been established to date. In this work, a search for homologs of the hypothetical amino acid sequence of the gene product through databases, as well as an analysis of the homolgs, was performed; the distribution of secondary structure elements was theoretically predicted; and the tertiary structure of the RibT protein was proposed. The *rib*T gene nucleotide sequence was amplified and cloned into the standard high-copy expression vector pET15b and then expressed after induction with IPTG in *E. coli *BL21 (DE3) strain cells containing the inducible phage T7 RNA polymerase gene. The *rib*T gene expression was confirmed by SDS-PAGE. The protein product of the expression was purified by affinity chromatography. Therefore, the real possibility of RibT protein production in quantities sufficient for further investigation of its structure and functional activity was demonstrated.

## INTRODUCTION

The main stages in the riboflavin biosynthesis in* Bacillus subtilis *cells have been elucidated previously. This process turns out to be controlled by two regions of the genome: the *rib *operon and the bifunctional flavokinase/FAD synthase gene, *rib*C, which is part of the *tru*B-*rps*O operon [1, 2]. The *rib *operon, which controls the overall pathway of riboflavin production, starting with guanosine-5’-triphosphate (starting precursor), consists of five nonoverlapping genes. These are four consecutive structural genes:* rib*G (encodes bifunctional aminopyrimidine deaminase/ uracil reductase), *ribB *(riboflavin synthase gene), *rib*A (GTP cyclohydrolase gene), *rib*H (lumazine synthase gene), as well as *rib*T, the operon’s terminal gene, whose function has not been determined so far. Furthermore, this operon contains three regulatory elements: the *rib*O regulatory region with the major promoter P1 and two additional internal promoters P2 and P3.

Previously, we determined the relative functional activity of all three operon promoters [3]. In this case, a paradox came to light: the P2 and P3 promoters, when tested separately within corresponding fragments of the operon, differed considerably in their transcriptional activity. The activity of the P3 promoter, which regulates *rib*T gene transcription, exceeded several times the major P1 promoter activity. The P2 promoter, on the contrary, was tens of times weaker than P1. However, neither P2 nor P3 is regulated by flavins, but it is known that when the entire *rib *operon is transcribed under the major P1 promoter control, all elements of the operon are transcribed in concert to form a polycistronic mRNA [4]. And this is despite the presence of several promoters that differ in their transcriptional activity and regulatory mechanisms.

The *rib*T (*ypz*K in other nomenclatures) gene function still remains quite unclear. Indirect indications have been obtained that mutations in the *rib*T gene affect the *rib *operon activity and riboflavin accumulation. For example, Perkins *et al*. [5] demonstrated that the *rib*T gene inactivation does not lead to riboflavin auxotrophy, but it significantly reduces the riboflavin yield in producer strains. This suggests that the *rib*T gene function is important for the riboflavin biosynthesis, but it may become limiting at maximum intensity of riboflavin biosynthesis.

Therefore, elucidating the function of a *rib*T gene product may provide additional opportunities for the development of commercially promising superproducers of riboflavin, which is one of the most important vitamins.

**Fig. 1:**
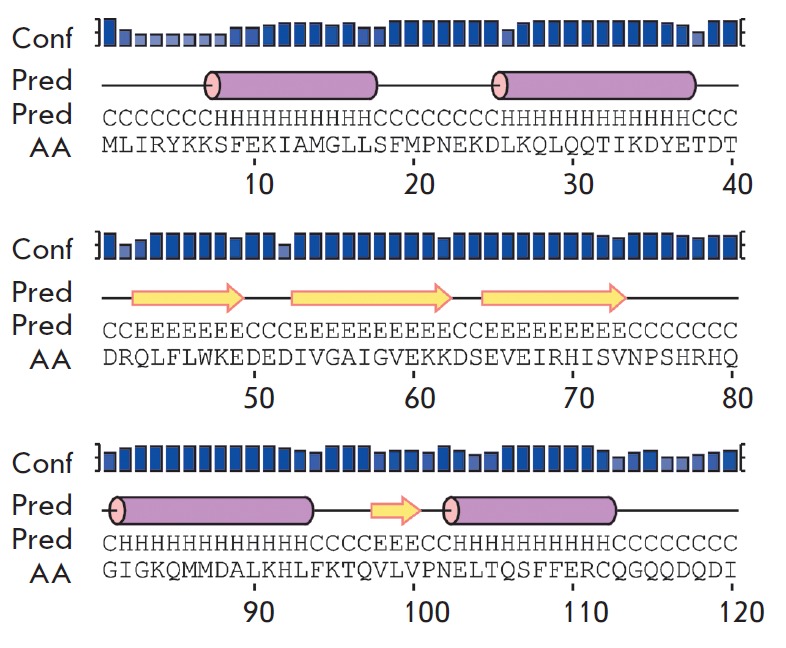
Secondary structure elements predicted for the ribT gene product

Based on the known nucleotide sequence of the *rib*T gene, the amino acid sequence of its protein product was deduced. The hypothetical protein consists of 124 amino acids and has a molecular weight of 14.5 kDa. The prediction of the regions that form, with a high probability, the elements of the secondary structure (*Fig. 1*) was made using the PSIPRED service [6].

**Fig. 2:**
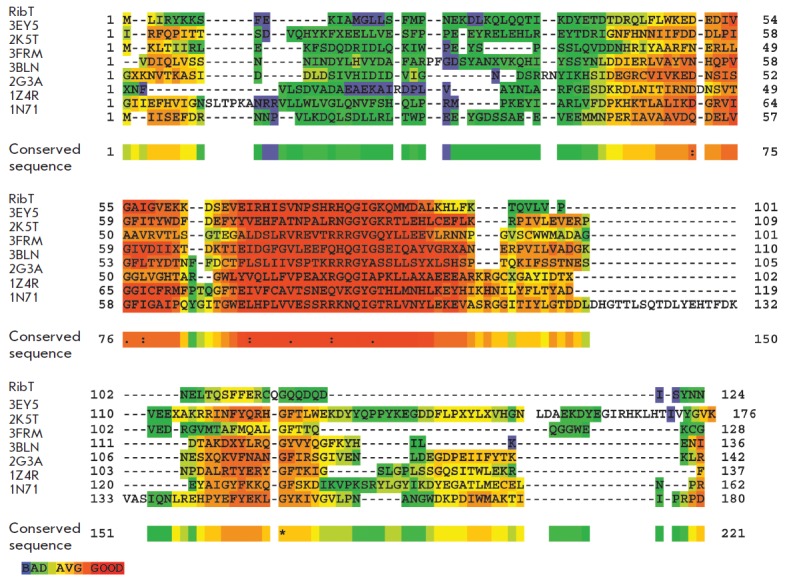
Multiple alignment of the amino acid sequence of the ribT gene protein product to the assumed homologs (the description of the color scheme is provided at http://www.jalview.org/help/html/colourSchemes/clustal.html)

Then, based on this sequence, the search for homologs was carried out and a multiple alignment of the sequence of the *rib*T gene protein product was performed using the Clustal software [5] only among the proteins with structures deposited in the PDB [7] (*Fig. 2*).

**Fig. 3:**
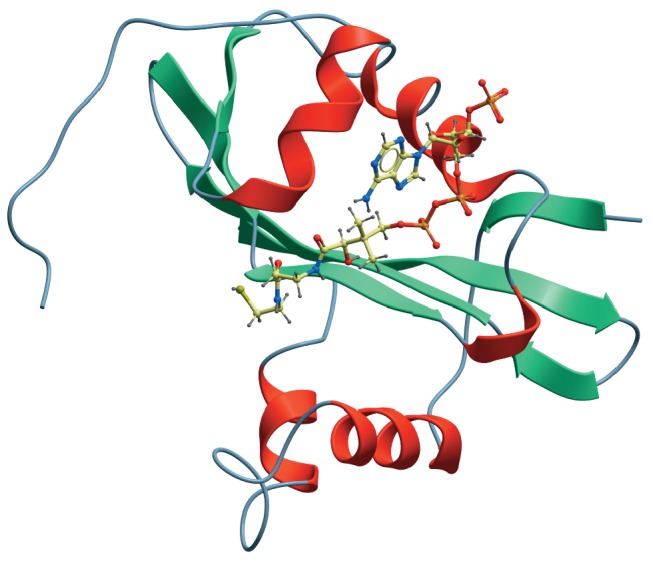
Proposed tertiary structure for the ribT gene product in a complex with acetyl-CoA

Of them, 1N71, 3FRM, 2G3A, 1Z4R, 3EY5, 2K5T, and 3BLN were selected, and based on homology, the structure presented in *Fig. 3* was built. Since acetyl CoA is present in the crystal structures of some homologous proteins, ligand docking to the hypothetical structure was carried out using the Molsoft ICM Pro software package [8].

Since most of the selected homologous proteins (except 3FRM, whose function is unknown) were acetyl transferases, the hypothetical *rib*T gene product could be assumed also to belong to this class of enzymes.

We assumed that the role of this gene’s protein product might be the acetylation of the N(5) atom of flavins that results in the production of their reduced forms and maintains a high transcriptional level for the *rib* operon. Previously, with our participation, the mechanism of transcriptional inhibition through the direct interaction of flavins with the leader sequence of mRNA was established [9]. The consistency of our assumption is confirmed by the fact that the inhibitory transcriptional interaction with the leader sequence of a sensory RNA is performed by the oxidized form of FMN [10]. Therefore, the acetyl reduction produced by the *rib*T gene product may be important for maintaining a high level of riboflavin synthesis. This assumption requires direct experimental verification.

This defines the objective of further research that is to perform the full cycle of *rib*T gene expression in a preparative mode, to produce sufficient quantities of the purified native protein product, and to test directly its functional (enzymatic) activity *in vitro*.

To test the possibility of expression, the *rib*T gene was amplified from chromosomal DNA of *B. subtilis* using the primers RibT10, 5’-CGCCATATGTTAATTCGTTATAAAAAATCGTTT- 3’, and RibT11, 5’-CGCCTC GAGTAATTATT GTATGAAATGTCTT - GATC-3’, (the oligonucleotides used in this study were synthesized at Evrogen). The first oligonucleotide is complementary to the proximal region, and the second is complementary to the distal region of the *rib*T gene. PCR was performed on a MyCycler thermal cycler (BioRad) according to the following scheme: first, cells were disrupted at 95 °C for 3 min, then 25 cycles of amplification were carried out that included DNA denaturation at 95 °C for 30 s, primer annealing at 60 °C for 30 s, and completion of DNA at 72 °C for 30 s. At the last stage, completion of DNA was done at 72 °C for 2 min. As a result, a fragment of 372 bp was synthesized that contained the structural region of the *rib*T gene flanked by the restriction enzyme recognition sites, NdeI and XhoI. After electrophoretic separation of PCR products, the desired DNA fragment was eluted from the gel using a GeneClean kit (Fermentas). The *rib*T gene was cloned into the pET15b high-copy expression vector containing the T7 phage promoter, which is inducible by isopropyl β-D-1-thiogalactopyranoside (IPTG), at the restriction endonuclease sites NdeI and XhoI. The PET15b vector comprises nucleotide sequences for His-Tag before the NdeI restriction enzyme recognition site and for the target site of thrombin. *E. coli* TGI strain cells were transformed with the resulting ligase mixture. Selection of transformants was performed in a LB agar medium containing ampicillin as a selection marker. Screening for recombinant clones was performed by PCR using the plasmid primers pT7P, 5’-TAATACGACTCACTATAGGGG-3’, and pT7T, 5’-GCTAGTTATTGCTCAGCGGT-3’. Plasmid DNA was isolated from the selected transformants, and the presence of insertion in hybrid plasmids was determined using a restriction analysis. These plasmids, designated as pET15b/*rib*T, were used to transform cells of the *E. coli *BL21 (DE3) strain containing the inducible T7 bacteriophage RNA polymerase gene.

**Fig. 4:**
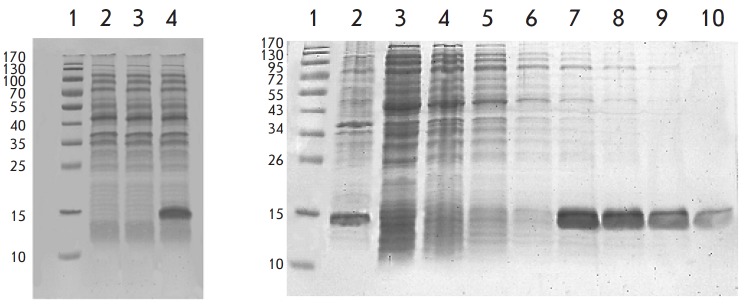
SDS-PAGE analysis of the ribT gene expression and recombinant RibT protein purification. A total protein fraction from E. coli BL21(DE3) cells. Line 1: a protein marker; line 2: a protein fraction from E. coli BL21(DE3) cells with the pET15b plasmid without ribT gene insertion; line 3: a protein fraction from E. coli BL21(DE3) cells with the pET15b/ribT plasmid, grown without IPTG addition; line 4: protein fraction from E. coli BL21(DE3) cells with the pET15b/ribT plasmid, grown in the presence of 1 mM IPTG. A protein fraction from E. coli BL21(DE3) pET15b/ ribT cells before and after affinity sorption on TALON® Magnetic Beads. Line 1: a protein marker; line 2: a total protein fraction from E. coli BL21(DE3) pET15b/ribT cells after IPTG induction; lines 3–6: protein fractions with proteins unbound to the affinity sorbent; lines 7–10: fractions of the His-Tag-labeled RibT protein successively eluted from the affinity sorbent with 300 mM imidazole

The RibT protein synthesis was induced by adding IPTG to the growth medium at a final concentration of 1 mM. The *rib*T gene expression was determined by polyacrylamide gel electrophoresis under denaturing conditions (SDS-PAGE) for a total protein from *E. coli* BL21 (DE3) cells containing the pET15b/*rib*T plasmid. As a control, the lysate of BL21 (DE3) strain cells containing the pET15b plasmid without the insertion was used (*Fig. 4A*).

An additional fraction of a protein with a molecular weight of approximately 14.5 kDa is observed in the cell lysate of the BL21 (DE3) strain containing the pET15b/*rib*T plasmid after IPTG mediated induction, which is consistent with the predicted molecular weight of the RibT protein (see above).

The His-Tag-labeled recombinant RibT protein was isolated using TALONR Magnetic Beads (Clontech, USA). Cells of the *E. coli *BL21 (DE3) strain containing the pET15b/*rib*T plasmid, grown in the presence of 1 mM IPTG, were harvested by centrifugation. The biomass was re-suspended in a buffer of the following composition: 20 mM sodium phosphate, pH 7.0, 300 mM NaCl, and 20 mM imidazole. The cells were disrupted by sonication and centrifuged at 14,000 rpm for 20 min. The supernatant was incubated with TALONR Magnetic Beads at 4 °C for 1 h. The resin was then washed with four volumes of the same buffer. Elution of the protein was performed with a buffer: 20 mM sodium phosphate, pH 7.0, 300 mM NaCl, and 300 mM imidazole. The eluted protein’s fractions were analyzed by SDS-PAGE electrophoresis (*Fig. 4B*). Lines 7–0 belonged to the target protein with a high degree of purity and a molecular weight of about 14.5 kDa, which is in good agreement with the theoretical prediction.

## CONCLUSIONS

The theoretical amino acid sequence and analysis of* rib*T gene product homologs in the PDB allowed us to perform molecular modeling and predicting of the possible three-dimensional structure of the protein. The* rib*T gene expression was experimentally implemented, and the possibility, in principle, to produce the RibT protein in quantities sufficient for further investigation of its structure and functional activity was proved.
